# Metastatic Hepatocellular Carcinoma Responsive to Pembrolizumab

**DOI:** 10.7759/cureus.631

**Published:** 2016-06-04

**Authors:** Phu Truong, Ahmad Rahal, K. James Kallail

**Affiliations:** 1 Cancer Center of Kansas, Wesley Medical Center; 2 Internal Medicine, University of Kansas School of Medicine-Wichita

**Keywords:** pembrolizumab, metastatic hepatocellular carcinoma, immune checkpoint inhibitors

## Abstract

Hepatocellular carcinoma (HCC) is an aggressive liver tumor that occurs with chronic liver disease. Surgical resection is the mainstay of therapy for localized disease whereas therapeutic options for advanced disease are limited. The innovative blockade of immune checkpoints with targeted immunotherapies, such as monoclonal antibodies against programmed death receptor 1 (PD-1), have shown promise in the treatment of solid malignancies. The PD-1 inhibiting antibodies, nivolumab and pembrolizumab prolonged overall survival in randomized trials in metastatic melanoma and advanced non-small cell lung cancer. This is a report of a 75-year-old male patient with metastatic HCC who was initially treated with the standard of therapy sorafenib. After failure of sorafenib therapy, pembrolizumab was started. There was a dramatic response to pembrolizumab with decrease in tumor size and drop in alfa fetoprotein. To the best of our knowledge, this is the first case report of metastatic HCC responsive to pembrolizumab after failure of sorafenib.

## Introduction

Hepatocellular carcinoma (HCC) is an aggressive tumor of the liver that usually occurs with chronic liver disease. HCC is frequently diagnosed late in its course with a median survival following diagnosis of approximately 6–20 months [1-2]. Extrahepatic spread is present at the time of diagnosis in approximately 5​–​15% of cases [3]. The most common sites of metastasis are lung, intra-abdominal lymph nodes, bone, and adrenal glands. The mainstay of therapy for localized disease is surgical resection. In metastatic disease, therapeutic options are limited. Molecular targeted therapy with sorafenib monotherapy is the standard systemic treatment for advanced HCC [4].

Substantial progress has been made in the development of immunotherapy for the treatment of a variety of solid malignancies. The immune checkpoint proteins, programmed death receptor 1 (PD-1) and programmed death receptor ligand (PD-L1), play a major role in the immune resistance of tumor cells [5]. PD-1 is a checkpoint protein expressed on T cells, B cells, and NK cells. It is an inhibitory molecule that binds to PD-L1. PD-L1 is expressed on the surface of multiple tissue types, including both normal and cancer cells. The PD-1-PD-L1 interaction helps keep the T cells from attacking tumor cells in the body. We report a case of metastatic HCC that responded dramatically to the immunotherapeutic agent pembrolizumab, a PD-1 inhibitor, after failure of standard therapy with sorafenib. Informed consent was obtained from the patient for this study.

## Case presentation

**Figure 1:**
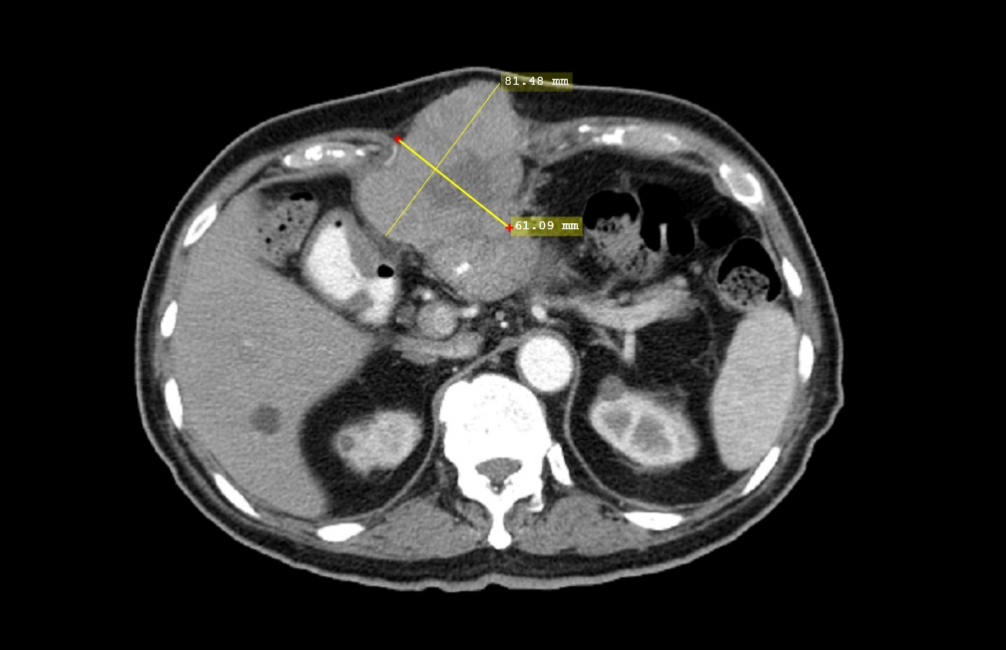
CT Abdomen Before Treatment With Pembrolizumab Axial contrast enhanced CT of the abdomen showing an 8 x 6 cm mass with central necrosis consistent with recurrent HCC.

A 75-year-old male patient presented with abdominal pain and with a past medical history significant for hypertension, gastroesophageal reflux disease, and anemia. An abdominal exam was positive for right upper quadrant tenderness. Laboratory findings revealed normal complete blood count, normal renal panel, but mildly elevated liver enzymes (aspartate aminotransferase (AST) = 66, alanine aminotransferase (ALT) = 38). A computerized tomography (CT) scan of the abdomen and pelvis showed a hyper-vascular mass in the left lobe of the liver. CT-guided biopsy of the mass showed HCC. The patient underwent exploratory laparotomy, total left hepatic lobectomy, and cholecystectomy. Active post-surgical surveillance with alpha fetoprotein (AFP) and CT scan of the abdomen was done for three years with no evidence of disease recurrence. Three years after initial presentation, the patient noticed a mass below the xiphoid process. AFP was elevated at 2751 mcg/L (normal 10–20 mcg/L). A CT scan of the abdomen showed a large anterior subxiphoid abdominal wall soft tissue mass measuring 8 x 6 cm with multiple small satellite lesions consistent with metastatic disease (Figure 1).

**Figure 2:**
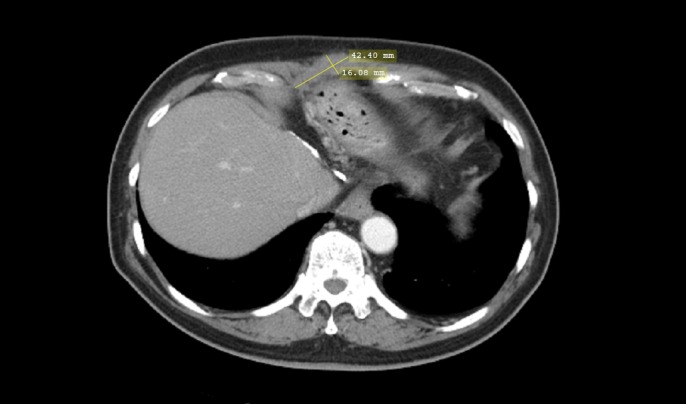
CT Abdomen After Treatment With Pembrolizumab Axial contrast-enhanced CT of the abdomen showing marked interval decrease in the size of the recurrent HCC measuring 2.2 x 1.6 cm.

Based on the elevated AFP and CT findings, the patient was considered to have relapsed metastatic HCC. He was started on standard therapy with sorafenib 200 mg orally twice a day for a week, then 400 mg orally twice a day. His course of treatment was complicated with hand-foot syndrome and the sorafenib dose was reduced from 400 mg to 200 mg twice daily. After five months of therapy with sorafenib, AFP increased from 2751 mcg/L to 8877 mcg/L and a repeat CT scan of the abdomen showed that the mass in the subxiphoid area was larger. With disease progression and failure of sorafenib, the patient was given the option of palliative care versus pembrolizumab on a compassionate use basis. After informed consent to individual therapy, off-label treatment with the anti-PD-1 antibody pembrolizumab (2 mg/kg every three weeks) was initiated. After six cycles of therapy, a CT scan of the abdomen showed a major decrease in subxiphoid mass size from 8 x 6 cm to 4 x 1.6 cm (Figure 2) and AFP dropped from 8877 mcg/L to 1.7 mcg/L. The patient tolerated therapy with pembrolizumab without adverse effects.

## Discussion

The innovative blockade of immune checkpoints with targeted immunotherapies, such as monoclonal antibodies against PD-1 and PD-L1, have shown great promise in Phase I clinical trials with activity seen in melanoma, non-small cell lung cancer, and renal cell cancer [6-7]. The PD-1 receptor is an inhibitory receptor and a negative regulator of T-cell effector mechanisms that limits immune responses against cancer [6]. Pembrolizumab is a highly selective, humanized monoclonal immunoglobulin against PD-1 that is designed to block the negative immune-regulatory signaling of the PD-1 receptor expressed by T cells, B cells, and macrophages [6]. Many tumors express high levels of PD-L1 to suppress antitumor immunity. PD-1 is expressed on antigen-stimulated T cells. When bound to the PDL-1, PD-1 results in T-cell suppression in peripheral tissues [8]. Anti-PD-1 works by inhibiting the interaction of PD-1 and PDL-1, thus reversing immune suppression induced by cancer cells [9].

HCC is typically an aggressive tumor that arises in the setting of underlying chronic liver disease in most cases. For patients with localized disease, surgery is the treatment of choice. When patients present with metastatic disease, therapeutic options are limited. Chemotherapy has not been used routinely for patients with advanced HCC, since HCC is considered to be a relatively chemotherapy-refractory tumor. Molecular targeted therapy with sorafenib is the standard systemic treatment for advanced HCC. Our patient was started on sorafenib, however, the tumor progressed and he failed standard therapy. Then, he was started on pembrolizumab with a significant improvement in AFP and subxiphoid mass. He tolerated pembrolizumab well with absence of colitis, hepatitis, pneumonitis, or pituitary dysfunction. After eight months of treatment, pembrolizumab not only caused shrinkage of tumor, but also improved survival in our patient with an Eastern Cooperative Oncology Group (ECOG) score of zero.

Our case revealed the potential efficacy of an anti-PD-1 antibody for treatment of metastatic HCC. An open-label, single-institution, non-randomized, single-arm study titled "Phase II study of Pembrolizumab (Keytruda) in Advanced Hepatocellular Carcinoma (HCC)" is currently evaluating the efficacy of pembrolizumab therapy in patients with advanced HCC. Results from this trial will contribute to determination of the benefit of PD-1 blockade in HCC.

## Conclusions

A promising avenue of clinical research in HCC is the use of immune checkpoint inhibitors. As far as we know, this is the first case report of advanced HCC responsive to pembrolizumab after failure of sorafenib therapy. Pembrolizumab and other immune checkpoint inhibitors could be a new developing therapy for patients with advanced HCC resistant to sorafenib.

## References

[1] Kew MC, Dos Santos HA, Sherlock S (1971). Diagnosis of primary cancer of the liver. Br Med J.

[2] The Cancer of the Liver Italian Program (CLIP) investigators (1998). A new prognostic system for hepatocellular carcinoma: a retrospective study of 435 patients. Hepatology.

[3] Uka K, Aikata H, Takaki S (2007). Clinical features and prognosis of patients with extrahepatic metastases from hepatocellular carcinoma. World J Gastroenterol.

[4] Llovet JM, Ricci S, Mazzaferro V (2008). Sorafenib in advanced hepatocellular carcinoma. N Engl J Med.

[5] Pardoll DM (2012). The blockade of immune checkpoints in cancer immunotherapy. Nat Rev Cancer.

[6] Hamid O, Robert C, Daud A (2013). Safety and tumor responses with lambrolizumab (anti-PD-1) in melanoma. N Engl J Med.

[7] Keir ME, Butte MJ, Freeman GJ (2008). PD-1 and its ligands in tolerance and immunity. Annu Rev Immunol.

[8] Francisco LM, Sage PT, Sharpe AH (2010). The PD-1 pathway in tolerance and autoimmunity. Immunol Rev.

[9] Okazaki T, Honjo T (2007). PD-1 and PD-1 ligands: from discovery to clinical application. Int Immunol.

